# Toward the remote monitoring of armed conflicts

**DOI:** 10.1093/pnasnexus/pgad181

**Published:** 2023-05-29

**Authors:** Valerie Sticher, Jan D Wegner, Birke Pfeifle

**Affiliations:** AI Governance Pillar, AI Singapore, innovation 4.0, 3 Research Link, 117602, Singapore; Center for Security Studies, ETH Zurich, Haldeneggsteig, 8092 Zurich, Switzerland; Institute for Computational Science, University of Zurich, Winterthurerstrasse 190, 8057 Zurich, Switzerland; Center for Security Studies, ETH Zurich, Haldeneggsteig, 8092 Zurich, Switzerland

## Abstract

The war in Ukraine has pushed the role of satellite imagery in armed conflicts into the spotlight. For a long time, satellite images were primarily used for military and intelligence purposes, but today they permeate every aspect of armed conflicts. Their importance in influencing the course of armed conflicts will further grow as progress in deep learning makes automated analysis progressively possible. This article assesses the state of the research working toward the remote monitoring of armed conflicts and highlights opportunities to increase the positive societal impact of future research efforts. First, we map the existing literature, categorizing studies in terms of conflict events that are covered, conflict context and scope, techniques, and types of satellite imagery used to identify conflict events. Second, we discuss how these choices affect opportunities to develop applications for human rights, humanitarian, and peacekeeping actors. Third, we provide an outlook, assessing promising paths forward. While much focus has been on high spatial resolution imagery, we demonstrate why research on freely available satellite images with moderate spatial but high temporal resolution can lead to more scalable and transferable options. We argue that research on such images should be prioritized, as it will have a greater positive impact on society, and we discuss what types of applications may soon become feasible through such research. We call for concerted efforts to compile a large dataset of nonsensitive conflict events to accelerate research toward the remote monitoring of armed conflicts and for interdisciplinary collaboration to ensure conflict-sensitive monitoring solutions.

Satellite images permeate every aspect of armed conflicts. While military and intelligence services have long used images from space for tactical and strategic purposes, the commercialization of satellites in recent decades has made them an invaluable source of data for an increasing range of organizations [(1), pp 225–250]. Far less intrusive than other forms of remote sensing, such as cameras mounted on drones, satellites often provide the only means to gather objective data from hard-to-access war zones even if such data only provides snapshots at a particular time. The war in Ukraine has pushed the role of satellite imagery in armed conflicts into the spotlight (2). Images of the buildup of Russian troops on the Ukrainian border in the lead-up to the Russian invasion triggered alarm bells in military headquarters (3–6) but were also featured in mainstream media [e.g. (7–9)]. Satellite images from the war were widely shared both in traditional media and on social media platforms, shaping public opinion and, by extension, the decisions of politicians regarding military support, sanctions, and the acceptability of a potential peace deal.

Beyond their highly visible use in media coverage, satellite images are also employed by a wide range of human rights, humanitarian, and peacekeeping actors to mitigate the impact of violence or support the resolution of armed conflicts (10–12). Traditional human rights organizations, like Amnesty International, have set up digital evidence labs (13) and join more loosely organized collectives, such as bellingcat, in using satellite images as a tool to verify military attacks and investigate war crimes in a variety of contexts including Darfur (14), Myanmar (15), and Ukraine (16). Humanitarian actors, like the International Committee of the Red Cross, utilize satellite images to verify reports, monitor critical infrastructure, and analyze hostilities during conflict situations (17). Peace operations of the United Nations and regional actors use satellite imagery to increase situational awareness (18), identify security threats (18), or monitor a ceasefire (19, 20).

In all these examples, image analysis is performed by humans. As a result of the labor-intensive nature of human classification, analysis tends to be ad-hoc and selective and predominantly conducted on conflicts that command international attention, such as the war in Ukraine. Driven by progress in deep learning and ever-increasing computing power, research aiming to automate parts of the analysis has advanced. Such research could enable a more systematic collection of fine-grained data points on conflict events, facilitating a variety of applications ranging from improved early warning systems to more efficient humanitarian assistance or even some form of conflict prediction.^1^ Yet, to date, endeavors to automate satellite image analysis have not permeated third-party efforts to mitigate or resolve armed conflicts beyond a small number of pilot projects (23, 24). In this perspective, we assess the state of the research toward the remote *monitoring* of armed conflicts, which involves regular near real-time screening of conflict events (rather than one-off mapping efforts). We look at its potential to support actors working to mitigate the effects of armed conflicts and discuss possible ways forward to bridge the gap between academic progress and societal impact through scalable and transferable solutions. We structure our analysis into three parts. First, we map the existing literature, categorizing the research in terms of types of conflict events covered, scope and conflict contexts, techniques, and types of satellite imagery used to identify conflict events. Second, based on research on structural damage—which we identify as the most promising subfield for influencing practice—we discuss how these choices affect the potential to turn academic proofs of concept into functioning applications with a positive societal impact. Third, we discuss implications and provide an outlook, assessing promising paths forward and the challenges they entail.

While much of the focus in research on remote sensing in armed conflicts has been on high spatial resolution imagery, we argue that freely available images with moderate spatial but high temporal resolution offer the most feasible path toward the systematic near real-time screening of large conflict areas, particularly if deep-learning models are used to combine information contained in optical and synthetic aperture radar images. Achieving high recall and precision in such models presents a significant challenge for the foreseeable future. We encourage organizations to prioritize the development of applications that limit the need to verify each individual prediction, such as systems that alert users based on specified geolocations. Finally, we call for concerted efforts to compile a large dataset of nonsensitive conflict events to accelerate research toward the remote monitoring of armed conflicts and interdisciplinary collaboration to ensure conflict-sensitive solutions.

## Mapping the field

To map the field, we conducted a systematic review of the literature focusing on the use of remote-sensing data in the context of armed conflicts. We aimed to include all relevant articles written in English in the past decade^2^ that were published in international peer-reviewed journals indexed by Web of Science or accepted at international scientific conferences.^3^ As our point of departure, we used three electronic databases with different ranking criteria.^4^ We conducted a simple search for articles including the keywords “satellite(s)” or “remote sensing” in combination with “war” or “conflict” and screened the top 100 results for relevance by title and abstract. We excluded archeological publications and articles that are primarily about the longer-term consequences of armed conflicts [see (25)], such as environmental degradation or the socioeconomic impact of wars. Through systematic forward and backward searches [see (22), p 104], we then expanded the body of relevant publications to 46. From those, we selected studies that highlight technical developments (29 articles) or use remote-sensing techniques to study conflict dynamics in specific cases (six articles), excluding other types of articles, such as sociological research [e.g. (26)] or reviews [e.g. (27, 28)].

This procedure set the stage for the first systematic review of the recent academic research on the use of remote monitoring in armed conflicts^5^ for which we used four main categorization criteria: types of conflict events covered, scope and conflict contexts, techniques, and types of satellite imagery used to identify conflict events. The purpose of these categorizations is two-fold. First, we aim to provide a broad overview, guiding interested readers on where to find information focusing on specific impacts of war visible from space (conflict events) or studies of specific locations (scope and conflict context). The second purpose of our categorization is to identify trends in current research approaches and to map these trends against what we see as the most promising path toward developing real-life applications. For that purpose, we distinguish techniques researchers use to detect conflict events and the types of satellite images they base their research on. We focus on these two considerations because they decisively shape the potential for scalable and transferable solutions. Note that all our categories are non-exhaustive and that there are other valid ways of organizing the information that may be better suited to a specific research need. From our mapping exercise, it becomes clear that research tends to be compartmentalized, even though wars are clearly not (29, 30). We use the subsequent sections to discuss how systems could become more scalable and transferable and thus more suitable for real-life applications in armed conflicts.

### Types of conflict events

Remote sensing can be used to identify a wide range of conflict events, from troop movement and the building of new trenches to damaged and destroyed infrastructure, flows of internally displaced populations, and changes in economic activities. While some events are immediately visible on satellite images, others appear with a temporal lag after kinetic conflict activity on the ground. Building on (25), we distinguish between conflict events that are immediately or almost immediately visible (within minutes to hours), those that have a short-term temporal lag (hours to days), and those with a mid-term lag (days to months).^6^ Table 1 offers a non-exhaustive typology of conflict events and the temporal lag with which they become visible, pointing to reviewed articles that discuss and identify these types of events.

**Table 1.** Types of conflict events, temporal lag, and articles focusing on these types of events. Source for the typology (25): own additions marked with ^a^.

Type	Examples	Temporal lag	Relevant articles
Kinetic activity^a^	Movement of troops^a^, movement of weaponry^a^, trenches^a^, landmines^a^, road blocks^a^	Immediate	(31)
Structural damage	Damaged or destroyed infrastructure (e.g. buildings, bridges), burned buildings, damaged electrical supply^a^	Immediate	(31–45); using nighttime light as proxy: (46–51)
Environmental damage	Oil spills, fires	Immediate	(32); using nighttime light as proxy: (46)
Environmental damage	Burned forests, burned oil wells	Short-term	(33)
Displacement	Movement of populations, informal settlements	Mid-term	(32, 52–62); using nighttime light as proxy: (63)
Decrease in economic activity^a^	Decrease in oil exploration^a^, decrease in tourism^a^	Mid-term	Using nighttime light as proxy: (51)

Two types of studies dominate research efforts in our area of study. The first is studies of structural damage, such as articles seeking to detect damaged or destroyed buildings [e.g. (34, 41)]. The second is studies of forced displacement, such as research identifying population flows [e.g. (62)] or refugee dwelling structures [e.g. (54)]. Detection methods are often developed for one specific type of event. This is why research presented in the articles tend to focus on one specific type, even if most conflicts display multiple conflict events (such as destroyed buildings and displaced populations). Some articles develop or apply research identifying multiple types of events, such as kinetic activity and structural damage (31), or structural damage, environmental damage, and displacement (32). Few of the selected studies focus on socioeconomic change or environmental damage, but there is a large subfield of research focusing on the longer-term environmental and socioeconomic consequences of armed conflicts (not included in this review).

Of note is the near absence of research on kinetic activity—such as the movement of weaponry or the building of trenches [see (31) for an exception]—despite the importance of these activities in influencing conflict dynamics. Because of its sensitive nature, reference data on kinetic activity is even harder to obtain than data on other conflict events; additionally, results from such research could be used for military purposes, especially if change detection is automated. Therefore, efforts in this area tend to be classified or not publicly available (1).

As indicated in Table 1, a subset of articles examines the use of nighttime light as a proxy for conflict events. These studies do not seek to identify specific instances of conflict events but instead assess the extent to which nighttime light reflects the prevalence of certain events and can thus inform conflict research or third-party engagement. Three of the selected articles (64–66) use nighttime light to identify larger conflict patterns, such as the occurrence and intensity of conflicts, and do not fit into the conflict-event typology outlined in Table 1.

### Conflict context and scope

The studies cover a wide variety of places, including countries in Northern Africa (36, 38, 44, 46, 47, 52, 53), Eastern Africa (52, 53, 55–57, 67), Middle Africa (53, 54, 56, 59, 61), Western Asia (31, 34, 35, 37, 39, 42, 43, 45–51, 58, 60, 63, 66), Southeastern Asia (32, 33, 40, 41, 56, 60), and the Caribbean (56).^7^ However, the variation becomes much narrower when we zoom in on a specific type of research, as our discussion of structural damage research below demonstrates.

Similarly, studies vary significantly in what they cover (see Table 2), but their scope tends to relate to the type of conflict event that is studied. Of the 14 articles that focus on the local level, 10 investigate displacement, often to identify individual dwelling structures or estimate the number of displaced people in an existing camp. Meanwhile, studies at the city level (eight out of nine) and the regional level (four out of five) primarily seek to identify structural damage,^8^ and all studies at the national and international levels use nighttime light as a proxy for conflict events, indicating that research beyond the identification of general patterns remains difficult on this scale.

**Table 2.** Scope of the analysis. Own categorization.

Scope	Description	Relevant articles
Local level	Studies covering one or several villages, local towns, city neighborhoods, or settlements	(36, 38, 40, 45, 52–58, 60, 61)
City level	Studies covering one or several cities	(31, 34, 35, 37, 39, 42, 43, 59) Nighttime light as proxy: (50)
Regional level	Studies covering one or several districts, provinces, or states	(32, 33, 41, 44); nighttime light as proxy: (66)
National level	Studies covering an entire country	Nighttime light as proxy: (48, 49, 51, 63)
International level	Studies covering at least two countries	Nighttime light as proxy: (46, 47, 64, 65)

### Techniques

Identifying conflict-related events in satellite imagery involves comparing images taken at different moments in time (change detection) or identifying objects within a single image (for example, tanks or tents in an informal settlement). While human manual classification has dominated analysis in the past, today the detection process is increasingly supported by automated analysis. For our review, we distinguish between traditional and artificial intelligence (AI)-based detection techniques. Human experts set the rules or define the procedure for traditional techniques, such as algebra-based methods [see (62), p 2]. Note that our use of the word “traditional” does not imply outdated; indeed, some of the reviewed studies use highly sophisticated signal processing techniques, such as interferometric synthetic aperture radar (InSAR) [e.g. (43)]. What distinguishes AI-based techniques from traditional techniques is therefore not the level of sophistication, but that algorithms (rather than humans) decide on the best approach, based on the models and data provided. In our understanding of the term, AI comprises traditional machine-learning methods, like random forests, maximum likelihood classifiers, and modern deep learning. We further distinguish between supervised AI-based detection, which establishes relationships based on human-labeled training data, and unsupervised AI-based detection, which establishes relationships based on patterns in multi-temporal data without the need for (manually) labeled data. Many promising approaches combine different techniques at different stages of the process.

As shown in Table 3, most of the reviewed articles use traditional methods, but research using supervised AI-based techniques is emerging (33, 34, 41, 42). Only one of the reviewed studies uses unsupervised AI-based techniques (55). We discuss the implications of such choices in the next section.

**Table 3.** Detection techniques.^9^ Own categorization based on (68).

Detection technique	Description	Relevant articles
Visual analysis	Humans identify likely conflict events through visual inspection	(56, 59)
Traditional techniques aimed at (partial) automatization	Experts defining specific rules and thresholds, algorithms identifying likely conflict events	(31, 32, 35–40, 43–53, 57, 58, 60, 63–66)
Supervised AI-based detection	Algorithms identifying likely conflict events based on training data	(33, 34, 41, 42, 54, 61)
Unsupervised AI-based detection	Algorithms identifying likely conflict events based on patterns in the data	(55)

### Types of satellite imagery

**Fig. 1.**
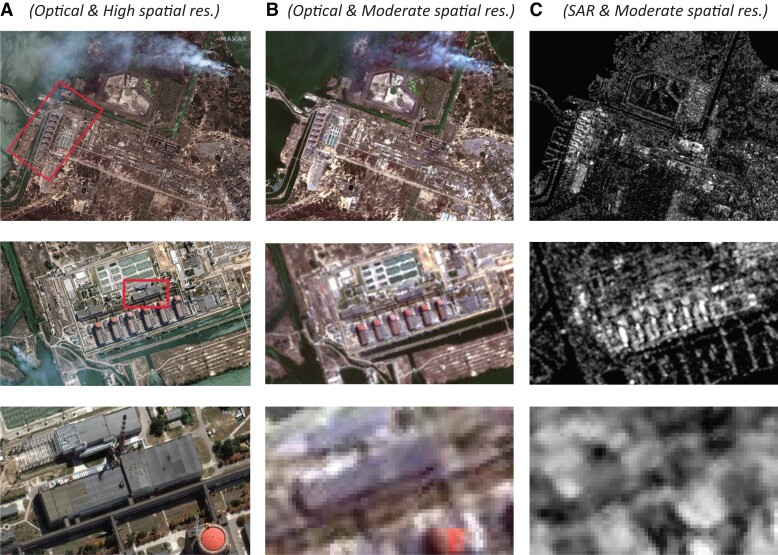
Site of the Zaporizhzhia nuclear power plant (Enerhodar) on August 29, 2022. The three rows of images show the same area with different zoom levels (red rectangles indicate the zoomed-in area). Image sources by column: Column A © 2022 Maxar (cluster 1, optical sensor), Column B Copernicus Sentinel-2 (cluster 3, optical sensor), and Column C Copernicus Sentinel-1 (cluster 3, SAR); Sentinel data processed by ESA.

Spatial resolution is the most prominent feature of satellite imagery, as it determines the size of the objects that humans can recognize in an image (see examples in Fig. 1). However, other features such as spectral or temporal resolution condition analysis and are gaining importance with progress in deep learning. In this article, we distinguish between imagery with high spatial resolution (here defined as a ground sampling distance [GSD] below 5 m), moderate spatial resolution (5–30 m GSD), and low resolution (more than 30 m GSD). We also distinguish between imagery that is tasked, that is, from constellations that can be requested to capture an image from a specific location and images from constellations with a regular revisiting cycle. As we discuss below, there are important differences between tasked and regular images, especially for multi-temporal analysis. Finally, images are available with different spectral resolutions, which refers to the wavelength intervals that are covered.

The images used in the reviewed articles fit into four broad clusters (see overview in Table 4). The first and most common type of imagery used for detection is commercial, archival tasked satellite images with high spatial resolution, such as images from the Maxar constellation. The second type, employed in only one study (40), is commercial imagery with high spatial resolution from a constellation with a regular revisiting cycle, in this case operated by Planet Labs. The third type, which is also common, is freely available satellite images with a regular revisiting cycle and moderate spatial resolution, such as images from the Sentinel satellite constellations of the European Space Agency (ESA). The fourth type is freely available images from sensors with a regular revisiting cycle but with low spatial resolution, such as images from the Visible Infrared Imaging Radiometer Suite instrument aboard the Suomi National Polar-orbiting Partnership satellites. This fourth cluster is relatively common, but it is used in the reviewed articles almost exclusively for nighttime light analysis [see (32) for an exception].

**Table 4.** Types of satellite imagery used to detect conflict events or patterns of events.^11^ Own categorization. Articles that use imagery from several clusters are marked with ^a^.

Cluster	Type of satellite data			Articles that use it for conflict event detection		
	Costs	Spatial Res.	Temp. Res.	Optical	SAR	Both
1	Commercial	High	Tasked	(33^a^, 36, 38, 45, 52–54, 56, 57, 60, 61)	[31^a^]	(58)
2	Commercial	High	Regular revisit	(40)		
3	Free	Moderate	Regular revisit	(33^a^, 37, 39, 41, 44, 55, 59)	[31^a^, 35, 43]	(32^a^, 42)
4	Free	Low	Regular revisit	(46–51, 63–66)		(32^a^)

**Fig. 2.**
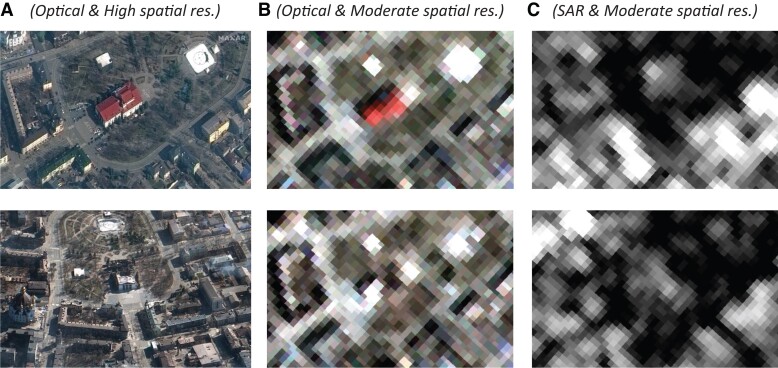
Mariupol drama theater before (row one) and after (row two) Russian shelling. Image sources by column: Column A © Maxar (cluster 1, optical sensor) March 14, 2022 (top) and March 19, 2022 (bottom), Column B Copernicus Sentinel-2 (cluster 3, optical sensor) March 14 and 19, 2022, and Column C Copernicus Sentinel-1 (cluster 3, SAR) March 16 and 24, 2022; Sentinel data processed by ESA.

Figures 1 and 2 illustrate some differences between the clusters based on example scenes from Ukraine. Satellite images from the site of the Zaporizhzhia nuclear power plant in Enerhodar show that, while moderate spatial resolution images can be useful to gain an overview of a scene, details at and beyond building level are difficult to recognize by the human eye (Fig. 1). Before and after images of the Russian attack on the drama theater in Mariupol show how difficult it is to recognize structural change in moderate (compared to high) spatial resolution imagery (Fig. 2).

Within our four clusters, we further distinguish between optical and synthetic aperture radar (SAR) imagery. As can be seen from Figs. 1 and 2, optical imagery is easier to interpret, especially for nonexperts. However, depending on the wavelength, radar imagery is much less affected by cloud coverage and not reliant on daytime light. As discussed in the next section, SAR images also contain information complementary to optical images that is of high value for AI-based techniques. However, most of the reviewed articles rely on optical imagery (29 studies), while the rest use SAR imagery (3 studies) or combine SAR and optical data (3 studies).

## Potential for applied use: the case of structural damage research

The choices outlined above, in particular the technique and type of imagery used to detect a conflict event, shape how easily proofs of concept can be turned into working applications that support the ongoing work of human rights and humanitarian and peacekeeping actors. We discuss this relationship by focusing on the example of research on structural damage, which we believe offers the most promising avenue for supporting a wide range of actors working to mitigate the effects of armed conflict. Research in other areas, such as displacement, also has a high potential to support the work of third-party actors but tends to serve more narrow and specific purposes, such as facilitating camp management or resource allocation. Of the reviewed articles, 21 focus on structural damage; among those, 15 seek to identify damaged or destroyed infrastructure, and six use nighttime light to identify patterns of destruction.^10^

We specifically consider how the technique and type of satellite imagery affect the scalability and transferability of a proposed approach as well as the resources required to set up a system, run it, and interpret its findings. In our case, a scalable solution is one that easily translates to detection of the same conflict event in other areas in the same or a highly similar conflict context. A transferable solution can be adapted for use in a different conflict context or for different types of events. For example, most research efforts to identify structural damage focus on damage and destruction caused by heavy artillery or air strikes in cities in Syria and in Mosul, Darfur, and the Gaza Strip (35–39, 42–45). Scalability indicates the extent to which such research can be useful in other places with other types of damage, including that inflicted by nonconventional forces.

### Scalability and transferability

Structural damage is often immediately visible in satellite imagery (25), depending on the extent of the destruction and the GSD of the satellite image. Researchers use change detection to identify instances of damaged or destroyed infrastructure, manually or automatically comparing images acquired before and after an attack. Visual inspection by humans transfers easily but does not scale. By contrast, traditional techniques can scale but tend to work well only for a specific purpose and place. They require rule and threshold adaptations with expert inputs for each new context or event, which is a major disadvantage of techniques such as object-based image analysis.

AI-based techniques, although often more complex and resource intense to set up, have a high potential for scaling and transfer. In the case of supervised AI-based change detection, transferability usually depends on new training data and, more importantly, reference data, which often remain scarce. Meanwhile, unsupervised methods learn from data in each new context or situation and do not require human-labeled reference data for training. Yet, while they are increasingly apt at producing accurate change maps, they are less suited to identify specific types of conflict events and the likelihood that they will occur, requiring human interpretation or a combination with other, supervised techniques. To avoid false discoveries that may possibly mislead policy making, AI research in remote monitoring of armed conflicts needs to closely collaborate with the domain experts and stakeholders that have a deep understanding of the specific conflict. In addition, quantitative evaluation of the proposed method needs to be implemented to understand the type of errors that occur and to make those transparent to decision makers.

**Fig. 3.**
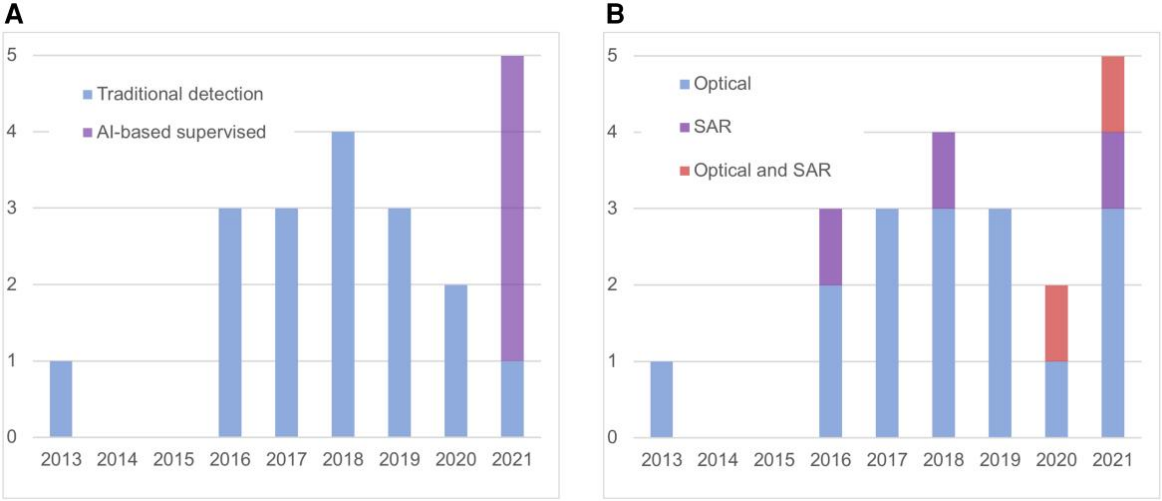
A: articles by year of publication (structural damage studies only). Own categorization. B: articles by type of sensor (optical vs SAR, structural damage studies only). Own categorization. See Table S1 for a detailed list.

As evident in Fig. 3A, research on structural damage has built up steam since 2016. Most studies use traditional change detection, but newer research (33, 34, 41, 42) increasingly employs supervised AI-based techniques, promising a shift to more transferable and scalable solutions. These studies combine multiple techniques (34, 42) or SAR and optical imagery (42) to increase the accuracy of structural damage prediction (Fig. 3B). One interesting innovation is the use of temporal smoothing, which builds on the assumption that as long as war rages, buildings tend not to be immediately reconstructed, which is information that can be used to validate predictions about changes detected in previous before–after comparisons of images (34).^12^

### Types of images

Compared to research on displacement, which is dominated by high spatial resolution images from cluster 1, research on structural damage is more varied in terms of the imagery used for detection. These choices have important implications for the resources needed to run a system and interpret and use its findings.

#### Practical considerations for running a monitoring system

Acquiring suitable satellite images to continuously screen for conflict events has important cost implications. High spatial resolution images, such as those in clusters 1 and 2, become very expensive if an organization tasks a constellation to cover a specific area frequently with newly acquired imagery. For example, according to estimates in (2, p 1), weekly high spatial resolution imagery covering all of Ukraine would add up to approximately USD 707 million per year. Archival images are much cheaper, and in some instances, satellite providers offer free access to images for research purposes. This makes it possible to train an algorithm and produce a proof of concept; however, to translate it into a functioning system—particularly one that offers regular, near real-time screening of entire conflict regions—images are needed on a repeated basis, quickly making the costs of purchasing commercial images prohibitive. Screening solutions with high spatial resolution imagery may be interesting for governments or regional and international organizations with access to constellations run by their member states, but they are less viable for nongovernmental organizations.

High spatial resolution images also tend to be larger in file size and cover much smaller areas than images with moderate or lower spatial resolution. The smaller coverage area means that more images from clusters 1 and 2 are required to cover the same area as images from clusters 3 and 4. Besides acquisition costs, this rapidly translates into massive demands for data download and processing. Although the rise in cloud solutions has reduced the need for expensive in-house infrastructure, implementing processing-hungry solutions can again quickly become expensive and have a large carbon footprint.

#### Recall and precision to use a monitoring system's findings

Aside from the resource requirements for running a system, organizations need human resources to interpret the data and use the findings. For some applications, such as the prioritization of humanitarian relief or situational awareness, an overall reliable estimate of the scope of the damage and where it occurs may be sufficient. In these cases, avoiding systematic biases, such as underreporting destruction in rural areas, may be as important as reducing false positives. For other applications, such as investigations of potential war crimes, organizations need to know with very high certainty that an event has indeed occurred. Such requirements are at odds with AI-based techniques because these techniques only provide likelihood estimates and no definitive certainty regarding an event. Moreover, although they can provide well-calibrated uncertainties along with the model outputs, AI-based techniques may suffer from model biases caused by, for example, biased training data.

Whenever a high level of certainty is needed, outputs from AI-based models need to be verified by human experts through visual analysis of high spatial resolution imagery or, where possible, ideally through ground truth. However, such human verification can be very labor intensive, especially when data samples are highly imbalanced, as tends to be the case in armed conflicts. Even when war rages, the percentage of buildings destroyed within a few days is usually very low compared to unchanged, intact buildings; consequently, even a low false-positive rate can translate into an unmanageable number of model predictions that have to be verified (34). To demonstrate the scope of the problem, let us imagine a system that correctly detects all damaged structures, but incorrectly labels 5% of undamaged structures as damaged, and a conflict area with an average of one in 1,000 buildings destroyed during a monitoring cycle. Even with perfect sensitivity and such a low false-positive rate, human experts would need to verify 51 instances to find one damaged structure in this scenario. If the system only recognizes 80% of damaged structures, and incorrectly labels 20% of undamaged structures as damaged, the average number of instances to be verified to find one damaged structure increases to 251. The more frequently analysis is performed, and the wider the area that is monitored, the more investment should be made to improve precision (the fraction of true positives among all the flagged possible events) and recall (the fraction of true positives from all events that should have been flagged), especially if users need to verify each event to be able to use the information.

Spatial resolution has long been the primary image characteristic shaping precision and recall, especially for events that are relatively small from space, such as building damage. However, while spatial resolution remains critical for detection, research in fields like environmental science shows that collecting evidence about objects of sub-pixel size (e.g. trees and cars) in images of 10 m GSD is indeed possible (70, 71), demonstrating that deep-learning models can pick up subtle changes that are not perceivable in the imagery by the human eye. Characteristics other than spatial resolution, such as spectral resolution, have also gained importance with deep learning's ability to deal with data that may not be immediately interpretable by humans (72, 73).

In assessments over time, temporal resolution can play a crucial role [(29), pp 228–229]. As noted above, subsequent images from the same place can be used for temporal smoothing (34), and higher temporal resolution can thus increase the performance of AI-based techniques. In the case of multispectral, optical imagery, high temporal resolution increases the chance of cloud-free views of the ground, which is especially important in cloudy regions or seasons (e.g. in the tropics). Higher temporal resolution also means that analyses can be performed more regularly or faster after an event (such as an air strike) and help organizations pin down the timeframe within which a conflict event has likely taken place. This can be important information to hold conflict actors accountable for atrocities as the same conflict area may be under the control of different actors at different times [(33), p 30].

Images from constellations with regular revisiting cycles (clusters 2–4) are usually available with high temporal resolution, whereas archival tasked images do not have any regular temporal resolution and may or may not be available for a specific period, depending on the interest of other actors in the area at the time. In theory, organizations could task a constellation from cluster 1 to cover a conflict area regularly, but as discussed above, this usually comes at exorbitant costs. Images from tasked constellations may also be taken at different times of the day and from different angles, posing additional challenges for automated change detection.

#### The promise of SAR imagery

While optical sensors record the reflectance of the sunlight at the earth's surface, synthetic aperture radar (SAR) sensors actively emit a microwave signal. They can thus acquire images independent from the sunlight and through clouds, and the slant-range mapping principle of SAR can detect subtle changes invisible directly from an optical, nadir perspective. For example, a collapsed building with an intact roof could hardly be detected as damaged in an optical image directly from a nadir perspective, whereas evidence in SAR imagery would allow its identification.

Most studies on structural damage are conducted with optical imagery, but there has been an uptick in research based on SAR imagery in recent years (see Fig. 3B). This is in line with a broader trend in remote-sensing research and is largely due to the advent of AI-based techniques, which can make use of information contained in SAR that may not be interpretable even by human experts [(67), p 2]. For a long time, using SAR imagery as part of automated analysis has been challenging for several reasons [for a discussion, see (67), p 5], including one specific property of the SAR image generation process: the intensity value of a SAR image pixel is the sum of all signals returned to the satellite within a resolution cell. Small (often metal) objects within a cell can drastically increase the amplitude of a backscattered signal (75). Neighboring cells without such strong scatterers would have much lower intensity, ultimately leading to dark and bright image intensities directly next to each other in the SAR image. This property is the so-called speckle effect, which looks like granular noise in the image texture (75). This effect is often considered undesirable but is indeed a true signal and of high value for AI-based change-detection efforts. Since subtle damages of objects within one resolution cell of a SAR image result in potentially large changes in the overall signal sum (i.e. image intensity at a specific pixel), it is possible to identify changes of sub-pixel size. To what sub-pixel level this can be pushed remains an empirical question, offering an exciting avenue for future research,^13^ including potentially making use of interferometric SAR techniques (75).

## Discussion and outlook

All the research choices discussed above involve trade-offs, especially in the selection of appropriate satellite imagery. High spatial resolution images make it easier to identify changes but require considerable processing power, are sometimes not available in high temporal resolution, and may be prohibitively expensive when used for screening purposes. Moderate spatial resolution images are freely available in high temporal resolution but make it harder to detect conflict events. Nonetheless, we believe that recent progress in deep learning has tilted the balance in favor of moderate-resolution imagery: deep-learning algorithms can pick up on subtle changes and information that are not interpretable by humans, making change detection at the sub-pixel level possible. Combining optical images with complementary data from SAR sensors offers a promising path forward. Particularly valuable for such endeavors are the Copernicus constellations operated by the European Space Agency, which offer freely available SAR (76) and multispectral optical images (77) covering the entire globe at regular intervals.

AI-based techniques are especially suited to combine data points from different sources and, as discussed above, scale and transfer much better than traditional methods. One technique that has yielded promising results in other fields but has not been used in any of the reviewed articles on armed conflicts is the use of so-called Siamese convolutional neural networks (CNNs) (78–81). Siamese CNNs take a pair of co-registered satellite images as input, pass each separately through a feature encoder to extract the most important evidence, and finally compare this evidence per pixel. The output is a change map that classifies the region of interest into unchanged and changed areas at the pixel level. This can be extended to more classes—for example, the change class can be further subdivided into “armed conflict changes” and “changes”. A major advantage of such a learning-based approach is that because all differences (i.e. damages caused by armed conflict) are learned end to end, the method is scenario agnostic. If new conflict scenarios emerge in regions that have not been shown to the model during the training phase, the existing model can be fine-tuned on a small set of manually annotated events of the new scene to produce accurate armed conflict monitoring maps. The method and software remain unchanged; the existing approach simply has to be retrained once more to accurately digest the new scene layout due to, for example, different landscapes and possibly new types of conflict evidence.

The scarcity of reference data on conflict events remains a constraining factor for such research. One way to overcome these challenges could be to use similar data, such as data on structural changes caused by natural disasters, which is much more widely available [e.g. (82)], to pretrain deep-learning models across different geographical regions, then retrain the models for armed conflict events and fine-tune them for selected conflict contexts. However, even with such an approach, sufficient reference data will be crucial to pushing the research agenda forward. We therefore call on organizations working with remote-sensing data in the context of armed conflicts to contribute to progress in the field by making any data that is not sensitive available for research purposes. Close collaboration between researchers and practitioners can ensure that data are shared in a way that does not endanger any vulnerable populations [see discussion in (19), p 4] and facilitates processing through learning-based techniques.

### Feasible applications

Organizations seeking to enhance their use of satellite images to mitigate the effects of conflict violence should ask themselves a series of questions before setting up a new monitoring system. These include how often and at what scale they intend to use satellite images to support their work, ranging on a spectrum from a one-off mapping of a limited area to continuous screening of entire conflict areas. Similarly, they should consider what types of conflict event and what regions they are interested in, assessing how scalable and transferable a solution will need to be. Crucially, organizations must consider how fast a system should deliver and whether they are willing and able to invest human resources in a long-term project that may not yield any immediate results. The responses to these questions will help organizations identify the most promising approach in view of limited human resources. If results are needed fast and the frequency and scope of mapping is limited, organizations may be best served to continue relying on human annotation, at least until AI-enabled off-the-shelf applications are more readily available. If continuous screening of a range of conflict events in multiple locations is the goal, organizations should partner with researchers that can build AI-based monitoring systems, focusing on applications that are feasible given the remaining challenges associated with such systems.

One major challenge is the uncertainty inherent in the output generated by AI-based techniques. To be useful in practice, well-calibrated uncertainties as additional model outputs are essential [see (83)], requiring a specific model design for modern deep-learning techniques that calibrates uncertainties well. Instead of merely outputting the classes “unchanged”, “change due to natural causes”, and “change due to armed conflict” per pixel, for example, each event should be assigned a well-calibrated uncertainty value through combined insights from multiple deep-learning models (deep ensembles) (84) or by designing a deep-learning model that uses different model specifications within its architecture (implicit deep ensembles) (85). These uncertainty values can then inform an organization's follow-up actions.

Some third-party actors, such as peacekeeping missions, have a large presence of monitors on the ground, making it more feasible to follow up on each retrieved conflict event flagged with a high certainty level. However, many human rights and humanitarian actors, including large organizations, such as Amnesty International, Human Rights Watch, or the International Committee of the Red Cross, have only a few people on the ground in most conflict contexts, especially in conflicts that receive relatively little attention, which are precisely the places where automated remote monitoring could play an outsized role in helping to mitigate the effects of violence. These organizations may also find it too labor-intensive and costly to use high spatial resolution images to follow up on each individually flagged conflict event when hundreds of instances are potentially retrieved every few days.

In the near term, screening applications are therefore more likely to become possible if they limit the necessity of verifying each retrieved incident. We see three main types of applications that fit this criterion. The first are applications where gaining a sense of the scope and geographic and temporal distribution of the damage is sufficient. Better estimating the extent of the damage can, for example, increase the situational awareness of an organization present in a conflict zone or help plan humanitarian relief allocation. The second are applications where individual verification is needed for information to be helpful, but the number of instances that must be checked can be reduced through specified criteria. An organization can, for example, use regular screening to monitor critical infrastructure, specifying the geolocation of buildings that should be monitored and follow up only if an alert is raised in one of these locations. Third, remote monitoring can be used for early warning that triggers early action, especially in low-intensity conflicts that garner little international attention. Again, this would not require organizations to follow up on each retrieved incident, but alerts could be issued if geographical or temporal patterns and trends emerge. In each of these cases, combining remote-sensing models with data gathered from other sources, such as through automated social-media monitoring, could further support the decision-making of actors working in the fog of war.

Finally, researchers should consider the potential long-term consequences of efforts in this field of study. Satellite images have long been used as part of battlefield planning, and solutions that enable the near real-time screening of entire conflict areas could be turned into an instrument for tactical and strategic military purposes. The careful selection of the type of events that are monitored and the type of data that is shared publicly are important considerations to mitigate the risk of doing harm. Biases in detection can lead to biases in responses, with potential deadly effects for those in need of humanitarian aid. Beyond model or output biases, there is a risk that when some events become easier to monitor and quantify, they gain more attention while harder-to-monitor events (such as gender-based violence) may become increasingly neglected (20). This not only shapes third-party responses to violence but can also shift the behavior of conflict parties seeking to remain (literally) under the radar. Interdisciplinary approaches and close collaboration between researchers and the policy community are needed to ensure that we can harness the vast potential of remote monitoring of armed conflicts to alleviate suffering in wars without creating a host of new problems.

## Supplementary Material

pgad181_Supplementary_Data

## Data Availability

All data are included in the manuscript and the supplementary table.

## Footnotes

1 Note that conflict data availability is only one of several aspects limiting the accuracy of predictions [21, 22].

2 The search was conducted in May 2022 and includes articles published since 2012.

3 Note that papers accepted at international scientific conferences showcase ongoing research and may not have undergone the same level of quality control as papers published in peer-reviewed journals.

4 Web of Science, Google Scholar, and Altmetric Explorer.

5 [28] offers a scoping review of related literature focusing on how remote sensing can complement international peace and security activities.

6 Note that we exclude articles focusing on the longer-term consequences of conflict (months to years).

7 Classification based on United Nations Geoscheme subregions.

8 Three studies (one at the city level and two at the regional level) also look at one other type of conflict event, such as displacement or environmental damage.

9 We classify studies as using AI-based techniques whenever they use any AI-based technique, even if they combine it with other (i.e. traditional) detection methods.

10 Four of the studies seeking to identify infrastructure damage and two of those using nighttime light to identify patterns of destruction also look at other conflict events.

11 Study (34) is not included in the categorization because the authors use high spatial resolution Google Images, which may include tasked images and images with regular revisiting cycles.

12 See also the econometric literature on space-time aggregation [e.g. (69)].

13 See (35), p 2319 for a discussion of the challenges related to sub-pixel analysis.
