# Supplementary material for: Toward the remote monitoring of armed conflicts
Source: PNAS Nexus. 2023 May 29;2(6):pgad181. doi: 10.1093/pnasnexus/pgad181 (PMC10291284; doi:10.1093/pnasnexus/pgad181)
Supplement: pgad181_Supplementary_Data [file pgad181_supplementary_data.docx]

| **Authors** | **Year** | **Type of research** | **Type of Conflict Events** | **Conflict Events** | **Scope** | **Region (UN Geoscheme)** | **Technique type** | **Satelite image cluster^[[1]](#footnote-1)^** | **Optical or SAR** |
| --- | --- | --- | --- | --- | --- | --- | --- | --- | --- |
| Aung | 2021 | Conflict Study | Structural damage, environmental damage | Burned land, demolition of human settlements | Regional | South-eastern Asia | AI-based supervised | Clusters 1  and 3 | Optical |
| Boloorani et al. | 2021 | Technical | Structural damage | Damaged and destroyed urban areas | City/cities | Western Asia | Traditional detection | Cluster 3 | SAR |
| Braun | 2018 | Technical | Structural damage | Damaged and destroyed buildings | City/cities | Western Asia | Traditional detection | Cluster 3 | SAR |
| Checchi et al. | 2013 | Technical | Displacement | Displaced populations | Local | Southern Asia, Middle Africa, Eastern Africa, Caribbean | Visual analysis | Cluster 1 | Optical |
| Coscieme et al. | 2017 | Conflict Study | Other (proxy) | Occurrence and intensity of conflicts | International | Global | Traditional detection | Cluster 4 | Optical |
| Fakhri and Gkanatsios | 2021 | Technical | Structural damage | Damaged and destroyed buildings, loss of settlement | City/cities | Western Asia | AI-based supervised | Cluster 3 | Both |
| Friedrich and Van Den Hoek | 2020 | Technical | Displacement | Refugee settlements | Local | Eastern Africa | AI-based unsupervised | Cluster 3 | Optical |
| Ghorbanzadeh et al. | 2018 | Technical | Displacement | Tents and buildings in refugee settlements | Local | Middle Africa | AI-based supervised | Cluster 1 | Optical |
| Ghorbanzadeh et al. | 2021 | Technical | Displacement | Dwellings in a refugee camp | Local | Middle Africa | AI-based supervised | Cluster 1 | Optical |
| Jenerowicz et al. | 2019 | Technical | Displacement | IDP settlements, refugee camps | Local | Northern Africa, Eastern Africa | Traditional detection | Cluster 1 | Optical |
| Jiang et al. | 2017 | Conflict Study | Decrease in economic activities, structural damage (proxy) | Damage of urban infrastructure, decrease in oil exploration | National | Western Asia | Traditional detection | Cluster 4 | Optical |
| Kahraman et al. | 2016 | Technical | Structural damage | Damaged buildings | Local | Western Asia | Traditional detection | Cluster 1 | Optical |
| Knoth and Pebesma | 2017 | Technical | Structural damage | Destructed dwellings | Local | Northern Africa | Traditional detection | Cluster 1 | Optical |
| Knoth et al. | 2018 | Technical | Structural damage | Destroyed dwelling structures | Local | Northern Africa | Traditional detection | Cluster 1 | Optical |
| Levin et al. | 2018 | Technical | Structural damage (proxy) | Damaged infrastructure | International | Northern Africa, Western Asia | Traditional detection | Cluster 4 | Optical |
| Levin et al. | 2019 | Conflict Study | Structural damage (proxy), environmental damage (proxy) | Infrastructure damage, fires | International | Northern Africa, Western Asia | Traditional detection | Cluster 4 | Optical |
| Li and Li | 2014 | Technical | Displacement (proxy) | IDP population | National | Western Asia | Traditional detection | Cluster 4 | Optical |
| Li et al | 2013 | Technical | Other (proxy) | Onset of conflict, ceasefires | International | Global | Traditional detection | Cluster 4 | Optical |
| Li et al. | 2017 | Technical | Structural damage (proxy) | Damage and reconstruction of electricity supply | national | Western Asia | Traditional detection | Cluster 4 | Optical |
| Li et al. | 2018 | Conflict Study | Structural damage (proxy) | Damaged electric supply | National | Western Asia | Traditional detection | Cluster 4 | Optical |
| Li et al. | 2015 | Conflict Study | Other (proxy) | Lack of access to electricity supply | Regional | Western Asia | Traditional detection | Cluster 4 | Optical |
| Lubin and Saleem | 2019 | Technical | Structural damage | Damaged buildings | City/cities | Western Asia | Traditional detection | Cluster 3 | Optical |
| Marx | 2016 | Technical | Structural damage | Destroyed urban buildings | City/cities | Western Asia | Traditional detection | Cluster 3 | Optical |
| Marx and Loboda | 2013 | Technical | Structural damage | Destroyed villages | Regional | Northern Africa | Traditional detection | Cluster 3 | Optical |
| Marx et al. | 2019 | Technical | Structural damage | Burned and burning villages | Local | South-eastern Asia | Traditional detection | Cluster 2 | Optical |
| Mueller et al. | 2021 | Technical | Structural damage | Destroyed buildings | City/cities | Western Asia | AI-based supervised | Clusters 1 and/or 2 | Optical |
| Pech and Lakes | 2017 | Technical | Displacement | Urban expansion | City/cities | Middle Africa | Visual analysis | Cluster 3 | Optical |
| Redmond | 2021 | Technical | Structural damage | Destroyed settlements and dwellings | Regional | South-eastern Asia | AI-based supervised | Cluster 3 | Optical |
| Ren et al. | 2020 | Technical | Displacement, structural damage, environmental damage | Fire, camp construction, razing of villages | Regional | South-eastern Asia | Traditional detection | Clusters 3  and 4 | Both |
| Shah et al. | 2020 | Technical | Structural damage (proxy) | Damage to electricity grids | City/cities | Western Asia | Traditional detection | Cluster 4 | Optical |
| Spröhnle et al. | 2014 | Technical | Displacement | IDP settlements | Local | Eastern Africa | Traditional detection | Cluster 1 | Optical |
| Spröhnle et al. | 2017 | Technical | Displacement | Different dwelling types in a refugee camp | Local | Western Asia | Traditional detection | Cluster 1 | Both |
| Tapete and Cigna | 2016 | Technical | Kinetic activity, structural damage | Military blockage, trenches, excavations, embankments, damaged buildings | City/cities | Western Asia | Traditional detection | Clusters 1  and 3 | SAR |
| Tiede et al. | 2017 | Technical | Displacement | Refugee camps | Local | Eastern Africa, Northern Africa, Middle Africa | Traditional detection | Cluster 1 | Optical |
| Wang et al. | 2015 | Technical | Displacement | Tents in IDP and refugee camps | Local | South-eastern Asia, Western Asia | Traditional detection | Cluster 1 | Optical |

Table S1: main attributes of studies included in the review section. Own categorization.

1. Cluster 1: commercial, archival tasked, high–spatial resolution images. Cluster 2: commercial, regular revisiting cycle, high–spatial resolution images. Cluster 3: freely available, regular revisiting cycle, moderate–spatial resolution images. Cluster 4: freely available, regular revising cycle, low–spatial resolution images. [↑](#footnote-ref-1)
